# The vertical symphony: how pitch perception shapes spatial and affective mapping across different countries

**DOI:** 10.1007/s10339-026-01364-2

**Published:** 2026-06-15

**Authors:** Fernando Marmolejo-Ramos, Carlos Tirado, Yuki Yamada, Kyoshiro Sasaki, José Hinojosa, Michał Parzuchowski, Magdalena Marszalek, Julian Tejada, Karel Rečka, Nadja Klein, Guillermo Briseño-Sánchez, Josef Kundrát

**Affiliations:** 1 College of Human Sciences and Culture, Flinders University, Adelaide, SA 5042 Australia; 2 Department of Behavioural Sciences and Learning (IBL), Linköping University, Linköping, Sweden; 3 Faculty of Arts and Science, Kyushu University, Fukuoka, Japan; 4 Faculty of Informatics, Kansai University, Takatusuki, Japan; 5 Instituto Pluridisciplinar, Universidad Complutense de Madrid, Madrid, Spain; 6 Departamento de Psicología Experimental, Procesos Cognitivos y Logopedia, Universidad Complutense de Madrid, Madrid, Spain; 7 Centro de Investigación Nebrija en Cognición (CINC), Universidad Nebrija, Madrid, Spain; 8 Department of Psychology, Center for Research on Cognition and Behavior, SWPS University, Sopot, Poland; 9 Department of Psychology, University of Sergipe, Aracaju, Brazil; 10 Department of Psychology, University of Ostrava, Ostrava, Czech Republic; 11 Scientific Computing Center, Karlsruhe Institute of Technology, Karlsruhe, Germany; 12 Department of Psychology, Pan-European University, Ostrava, Czech Republic

**Keywords:** Affective evaluation, Cross-national research, Cross-modal correspondence, Metaphorical mapping, Pitch height, Spatial associations

## Abstract

This study investigates the interrelationship between auditory pitch perception, spatial mapping, and affective evaluation in human cognition. We conducted three experiments to investigate the complex relationships between pitch height, spatial localization, and emotional valence. Experiment 1 (*n* = 63) revealed a non-linear relationship between pitch height and affective evaluation, with extremely high and low pitches receiving significantly less positive ratings than moderately high and low pitches. Experiment 2 (*n* = 70) demonstrated a strong and consistent spatial mapping of sounds along a vertical axis. As pitch height increased, sounds were systematically mapped from lower to higher spatial positions, supporting the idea of metaphorical mapping of pitch on the vertical dimension. Experiment 3 (*n* = 90) yielded inconclusive results regarding the separation of spatial associations in an implicit context. Collecting data from four countries enabled cross-national comparisons of these phenomena. Our findings enhance understanding of both universal and country-specific aspects of cross-modal associations between sound and space. These insights have implications for uncovering the cognitive mechanisms behind metaphorical mapping of sensory perceptions.

## Mapping pitch on space

### Vertical space

Pratt (1930) was the first to study how the perception of pitches differing in height could modulate spatial perception. In his pioneering study, he asked participants to locate on a vertical scale, going from the floor to the ceiling, the point of origin of various sounds. The sounds had different pitch heights and could originate randomly from five different locations on the vertical axis. The higher the pitch, the higher its point of origin was located on the scale. This cross-modal association between pitch and vertical space was confirmed in a follow-up study by Trimble (1934; see also Roffler and Butler 1968; Bregman and Steiger 1980), who showed that participants tend to localize higher pitched sounds higher in space compared to lower pitched sounds.

Congruency effects have also been observed with more automatic tasks. In Bernstein and Edelstein’s (1971; see also Evans and Treisman 2010) study, participants were asked to respond to visual stimuli that could appear in the upper or lower part of the screen while hearing high- or low-pitched sounds. Participants were faster when the position of the visual stimulus matched pitch height (high position—high pitch/low position—low pitch) compared to when it mismatched (high position—low pitch/low position—high pitch). Moreover, Melara and Marks (1990) have observed a similar congruency effect when the participants were asked to judge the meaning or the pitch’s height of the words “high” or “low” pronounced with a high- or low-pitched voice.

It has even been demonstrated that the spatial biases associated with pitch height can affect some aspects of everyday life activities (Parrott et al. 2015; but see Geronazzo et al. 2015). In their study, Parrott et al. (2015) demonstrated that participants were faster at identifying graphs that represented positive or negative slopes while listening to ascending or descending tones, respectively, compared to the opposite. On a related note, Lemaitre et al. (2017; see also Küssner et al. 2014) observed that participants tended to make upward or downward movements when imitating sounds with a high or a low pitch, respectively.

Beyond spatial mapping, auditory pitch is a fundamental carrier of affective information (Juslin and Laukka 2003; Juslin and Sloboda 2010). Research in psychoacoustics and music cognition has consistently shown that pitch height correlates with emotional responses, though the relationship is complex (Gabrielsson and Lindström 2010). Generally, higher pitches are associated with emotions of high arousal and positive valence, such as happiness, excitement, or joy (Hevner 1937; Juslin and Laukka 2003). Conversely, lower pitches are often linked to emotions of low arousal and negative valence, such as sadness, seriousness, or solemnity (Gundlach 1935; Scherer and Oshinsky 1977). These associations are prevalent in music, where composers often use pitch height to evoke specific emotional landscapes.^1^

However, this linear relationship is not absolute, as the affective response to pitch can be modulated by the interplay of other musical features like tempo and loudness (Gabrielsson and Lindström 2010; Ilie and Thompson 2006). More importantly, extreme pitches may break the simple “high = positive, low = negative” pattern. For instance, extremely high-pitched sounds can be perceived as piercing, alarming, or unpleasant (e.g., a shriek), while extremely low-pitched sounds can be experienced as ominous or threatening (e.g., a growl) (Scherer and Oshinsky 1977). This suggests a potential non-linear or curvilinear relationship between pitch height and emotional valence, where sounds in the moderate ranges are perceived as more pleasant than those at the perceptual extremes. Our study aims to explore this nuanced relationship directly. By systematically evaluating the emotional valence across a wide spectrum of frequencies, Experiment 1 investigates whether affective ratings indeed follow this non-linear pattern, providing a crucial affective dimension to the primary investigation of pitch-space mapping.

### Horizontal space

In addition to the vertical mapping of pitch, Mudd (1963) discovered that the pitch height was also mapped horizontally. In each trial, participants listened to two sounds that varied in pitch. They were asked to place a peg on a peg panel that would represent the position of the first sound (referent) and another peg that would represent the position of the second sound (target). When the pitch of the second sound was higher than the pitch of the first sound, the second peg was placed higher and more to the right compared to the first peg. It was the opposite when the second sound was of a lower pitch compared to the first sound. Mudd (1963) concluded that the pitch height must be represented multidimensionally, over the vertical (low vs. high) and the lateral (left vs. right) axes.

Investigating this lateral representation of pitch, Nishimura and Yokosawa (2009) used a task similar to a Simon task. In their study, participants were asked to judge, with their left or right hand, the color of a stimulus while they were listening to a high- or low-pitched sound presented to the left or the right ear. Responses with the right hand were faster and more accurate when they heard a high-pitched sound compared to a low pitched-sound. It was the opposite when the response was with the left hand. In their experiment, participants also responded to the color of a visual stimulus by pressing a left or right button while simultaneously hearing a high or low tone played to the left or right ear. The results showed two interesting phenomena: First, responses were faster when the tone came from the same side as the response (e.g., the tone in the right ear and the right button press). Second, regardless of which ear the tone was played into, high tones facilitated right-hand responses, whereas low tones facilitated left-hand responses. These findings show that even when participants were focused on the visual task, the auditory stimuli automatically activated the corresponding spatial associations, with high tones being mentally associated with the right hand and low tones with the left hand.

### Vertical vs. horizontal spaces for pitch height representation

Although evidence supports the representation of pitches along both the vertical and lateral axes, the strength of these representations appears to be unequal. Mudd (1963) was the first to notice that participants seem to prefer the vertical axis to represent pitch height. In his experiment described above, the second peg was placed farther from the reference peg on the vertical axis compared to the lateral axis. He concluded that the representation of pitch height on the vertical axis must be stronger than the representation on the lateral axis.

Rusconi et al. (2006) and Lidji et al. (2007) aimed to address this issue directly. They investigated the performance of musician and non-musician participants completing a judgment task on auditory features of sounds that differed in pitch height. Participants made judgments by pressing one of two keys arranged either vertically or horizontally. When the judgment task focused on pitch height (i.e., when pitch was task relevant), non-musician participants showed both horizontal (though only as a trend in Rusconi et al. 2006) and vertical congruency effects (i.e., faster and more accurate responses when high pitches corresponded to upper/right keys and low pitches to lower/left keys). However, when the judgment task focused on the type of musical instrument that played the sound (i.e., when pitch was task irrelevant), non-musician participants showed a vertical congruency effect only (see also Evans and Treisman 2010; for similar results on the vertical axis only). Musicians, on the other hand, exhibited congruency effects on both axes, even when pitch was task irrelevant.

Lega et al. (2014) observed a similar pattern in a line bisection task. Musicians and non-musicians were asked to indicate the middle of rods that varied in length while simultaneously listening to low- or high-pitched sounds. During this task, participants had to make judgments about the sounds they heard, with pitch being either task-relevant (judging if the tone was high or low) or task-irrelevant (judging the timbre of the tone). A first finding was that only musicians showed a bias according to the lateral representation of pitch. Second, this bias was greater when the pitch was task relevant compared to when it was not.

Repp and Knoblich (2007) also demonstrated that only musicians could modulate pitch perception through manual actions on the lateral axis. Participants had to press keys arranged from left to right or right to left, mirroring the movements on a piano keyboard. While they were carrying this action, an ascending or descending pitch was heard. Musicians tended to report a rising pitch more often when performing left-to-right actions than right-to-left actions, and conversely, reported a descending pitch more often when performing right-to-left actions than left-to-right actions. Repp and Knoblich (2007) concluded that the extensive training with a piano keyboard resulted in a mental mapping of the spatial coordinates of the notes corresponding to the different keys of the piano keyboard.

Therefore, it appears that the activation of the horizontal representation of pitch is less automatic than that of the vertical representation. Even further, musical training appears to play a role in this activation process. Musical training might enhance the ability to perceive pitch in a more automatic way. In this case, a higher consciousness of pitch height in musicians would allow the activation of its horizontal mapping, as it is the case when pitch height is explicitly processed by non-musicians. The habit of playing instruments where pitch rises from left to right, like for piano, might also contribute to the automatic activation of the horizontal representation of pitch (Repp and Knoblich 2007).

However, Nishimura and Yokosawa (2009) observed a congruency effect between pitch height and the horizontal space with non-musician participants, even though the pitch was irrelevant for the task. The authors hypothesized that it might be due to the fact that the school curriculum in Japan includes much more hours of musical training than in the other countries of the previous studies. However, to the best of our knowledge, there is no other study that replicated Nishimura and Yokosawa’s (2009) results and there is no study that directly addressed the difference between Japanese participants with participants from another culture, where the musical training at school is not as intensive.

Recent research by Dolscheid et al. (2023) sheds light on the cross-cultural variability in the representation of pitch, particularly regarding the association between spatial dimensions and auditory pitch. Dolscheid et al. (2023) investigated the developmental trajectory of linguistic and non-linguistic space-pitch associations in children who acquire languages with different pitch representations, such as Dutch (a height-pitch language) or Turkish (a thickness-pitch language). Their findings revealed that thickness-pitch associations were stronger across tasks than height-pitch associations, even among Dutch-speaking children not exposed to thickness-pitch vocabulary. Moreover, Turkish-speaking children exhibited reversed height-pitch associations. These results suggest that while thickness-pitch associations are acquired similarly across cultures, height-pitch associations are more susceptible to linguistic input. The study underscores the dynamic nature of space-pitch mappings and highlights the role of cross-cultural experiences in shaping music cognition.

The origin of pitch-space cross-modal mapping has long been debated, with early theories proposing either structural factors within the cochlea or the influence of auditory habits (Pratt 1930; Trimble 1934). Presently, Spence (2011) delineates three potential sources: structural, statistical, and semantic. Structural mechanisms posit innate cross-modal correspondences, while statistical and semantic mechanisms suggest learned associations influenced by multisensory experiences and language. Di Stefano (2022), on the other hand, proposes that the framework of the spatiality of sounds can be categorized into literal and metaphorical meanings. While literal spatiality refers to the external localization of a sound source in the physical environment, the metaphorical use of space describes a perceptual, non-material “sound space” or “musical space” where auditory objects are placed and perceived to move (Di Stefano 2022). This metaphorical mapping is rooted in a (pseudo)spatial phenomenology, where listeners consistently interpret the dynamic changes of acoustic features—such as pitch and loudness—as movements (e.g., “ascending” or “descending” melodies) through a spatial continuum. Such crossmodal associations between auditory and spatial features—linking sound to dimensions like elevation, size, and distance—suggest that the spatial dimension is fundamental to auditory perception, even though sounds are traditionally defined primarily as temporal entities (Di Stefano 2022). Recent research on infants and individuals from diverse cultures supports the existence of a vertical pitch representation irrespective of linguistic terms (Dolscheid et al. 2014; Parkinson et al. 2012).

However, studies on native speakers of Catalan, Spanish, and Farsi suggest that language influences the strength of cross-modal congruency effects (Fernandez-Prieto et al. 2017; Dolscheid et al. 2013). Training with linguistic expressions akin to those used by other cultures can modify these effects (Dolscheid et al. 2013). This implies that while innate representations of pitch may exist, they are likely modulated by language habits or statistical correspondences.

Regarding the “left position—low pitch/right position—high pitch” correspondence, the “octave illusion” effect suggests a connection with participants’ handedness (Deutsch 1974; Oehler and Reuter 2013). Structural and learned correspondences, as well as musical training, may influence this association (Rusconi et al. 2006; Lidji et al. 2007; Casasanto 2009). Nonetheless, the exact mechanism remains elusive, and further research, particularly with left-handed participants, is warranted (Beecham et al. 2009).

## Cognitive mechanisms of the pitch-space cross-modal mapping

It is interesting that some aspects of the pitch-space cross-modal correspondence appear to depend on a learning process, as various mechanisms have been proposed to explain how this works. Essentially, two contrasting views have been hypothesized, one based on the Mental metaphors theory (e.g., Beecham et al. 2009; Dolscheid and Casasanto 2015) and the other based the Polarity correspondence hypothesis (e.g., Cho et al. 2012). According to the Mental metaphors theory (Casasanto 2013; see also Santiago et al. 2011), when a concept is correlating with another, a mental representation corresponding to this correlation would be created. The correlation between these concepts could be linguistic (e.g., using polysemous words or using language patterns such as speaking of a high pitch in terms of something that flies: “She sang perfectly all the high-flying notes of the Mozart’s Magic flute.”) or based on sensory-motor experiences (e.g., seeing or feeling the Adam’s apple going up and down when producing high and low pitch sounds, respectively). Each mental representation corresponding to a specific correlation would be independent from the other mental representations.

On a different note, the Polarity Correspondence Hypothesis (Proctor and Cho 2006) suggests that each bipolar concept (such as height: low and high) would have an unmarked (or positive) pole and a marked (or negative) pole. The unmarked pole would be the by default pole and could be used to refer to the entire dimension of the concept whereas the marked pole could be used to refer to its polarity only. As an example, if we compare someone who asks: “How high was that note?” or “How low was that note?” In the first example, there are no expectation on the response (the response could be from very low to very high), but in the second example there is a clear expectation towards a low note response because the low pole is the marked pole and it refers only to the lower dimension of height. The congruency effects would arise between the pole values of each bipolar concept. The high pitch, high height and right laterality would be the unmarked poles whereas the marked poles would be the low pitch, low height and left laterality. The congruency effects between different conceptual dimensions would be interdependent because they would rely on a unique mechanism that do not take into account the semantic referent of each concept, only their polarities.

In order to disentangle which of these two mechanisms can explain better the spatial representations of pitch, each participant was asked to carry out two tasks, one on the vertical axis and another on the lateral axis. A cluster analysis allowed to test whether or not the participants who presented a greater crossmodal mapping on one axis also presented a greater congruency effect on the other axis. Such an effect would have favored the Polarity Correspondence Hypothesis (Proctor and Cho 2006). On the opposite, the Mental metaphors theory (Casasanto 2013) was predicting that the congruency effects observed on the vertical and the lateral axes should be independent one from each other.

Recent neuroscientific investigations provide insights into the underlying neural mechanisms of these cross-modal correspondences. McCormick et al. (2018) used functional magnetic resonance imaging to explore the neural basis of the pitch-elevation correspondence, finding involvement of multisensory attention mechanisms rather than semantic mediation or magnitude estimation. Complementing these neural findings, Parise et al. (2014) provided evidence for a statistical basis of the pitch–elevation correspondence, demonstrating that high-frequency sounds in natural auditory environments tend to originate from elevated positions, and that this ecological regularity is reflected in the filtering properties of the human outer ear, thereby shaping spatial hearing. Furthermore, Chiou and Rich (2012) demonstrated how auditory pitch could induce shifts in spatial attention, suggesting that these mappings may occur at a late stage of voluntary attention orienting. Additionally, Küssner et al. (2014) highlighted the role of musical training in enhancing the consistency of these cross-modal mappings, suggesting that experience and training of reading and writing in musical notation (the higher the pitch of notes, the higher they are positioned on the staff) could further refine these associations .

Beecham et al. (2009) and Dolscheid and Casasanto (2015) already showed some hints regarding the nature of this mechanism. Concretely, Beecham et al. (2009) used a within-participant design in order to compare the size of two different congruency effects: the “high position—high pitch/low position—low pitch” congruency effect and the “left position—low number/right position—high number” effect, also known as the SNARC effect (Dehaene et al. 1993). Beecham et al. (2009) observed that the congruency effects were independent from each other. The congruency effects between the pitch and another concept were observed only with the height, not the other marked concepts. Despite these intriguing results, the relationship between horizontal and vertical representations of pitch has not yet been studied. Consequently, the Polarity Correspondence Hypothesis (Proctor and Cho 2006) cannot be ruled out for these two congruency effects, which is why we decided to address this issue directly in this paper.

## Methods

### Participants

A total of 225 participants were recruited via convenience sampling and took part in the three experiments (see Table 1). All of them reported normal or corrected-to-normal vision, and normal audition. Studies were conducted according to the Declaration of Helsinki principles (WMA 2013) and received ethics approval from each participating country.

**Table 1:** Distribution of the number of participants by country, age, gender and handedness

Experiment	Country/Lab	n	Age		Gender		Handedness	
			Mean	SD	Female	Male	Left	Right
1	Japan	20	20.35	1.18	9	11	1	19
	Poland	23	22.65	5.94	13	10	1	22
	Spain	20	25.60	7.14	9	11	1	19
2	Japan	20	23.35	3.34	12	8	4	16
	Spain	30	21.13	2.43	18	12	3	27
	Sweden	20	26.75	5.27	14	6	1	19
3	Japan	30	20.57	2.19	13	17	3	27
	Spain	30	21.87	4.22	19	11	4	26
	Sweden	32	26.84	5.49	23	9	1	31

For the first experiment, one-third of the data was collected in Poland. For the subsequent two experiments, we used Swedish data instead due to the unavailability of our Polish partner and the prohibitive cost of alternative local recruitment, whereas Sweden offered a more viable and cost-effective sample

### Experimental design and implementation

The three experiments employ distinct methodological approaches. Experiments 1 and 2 are direct crossmodal matching tasks: participants explicitly map auditory stimuli onto an affective dimension (Experiment 1) or a two-dimensional spatial layout (Experiment 2), establishing whether and how consistent mappings emerge between pitch and another dimension. By design, these tasks do not measure crossmodal congruency effects in the interference-based sense used by Evans and Treisman (2010). Experiment 3, in contrast, employs a crossmodal congruency paradigm in which pitch is task-irrelevant and its concurrent influence on the speed of visual letter identification is assessed. We refer to Experiments 2 and 3 as the ‘explicit’ and ‘implicit’ tasks for convenience, reflecting the role of pitch in the participant’s response; these labels are not intended as equivalents of the ‘direct’ and ‘indirect’ test distinction used in the crossmodal congruency literature.

All experiments were conducted in psychological experimental laboratories using Psychtoolbox via MATLAB v8.6. Instructions were presented in the native language of the participants. The following are the specific procedures for each task.

#### Experiment 1—Sounds and mapping emotional space

Participants listened via headphones to six auditory stimuli in a randomized order that ranged from very low to very high frequency and rated the perceived emotional valence (degree of positivity or negativity) of each sound by moving a slider on a computer screen. Auditory stimuli consisted of complex tones with base frequencies of 40, 90, 202, 456, 1026, and 2308 Hz. Each tone included two higher harmonics: the first harmonic was one octave above the base frequency, and the second harmonic was a pure fifth above the first (−3 dB per harmonic). The base frequencies were approximately two pure fifths apart. This spacing was chosen to create perceptually distinct pitch levels spanning a wide range of audible frequencies, thereby maximizing sensitivity to potential cross-modal mapping effects across the pitch continuum.^2^

**Fig. 1:**
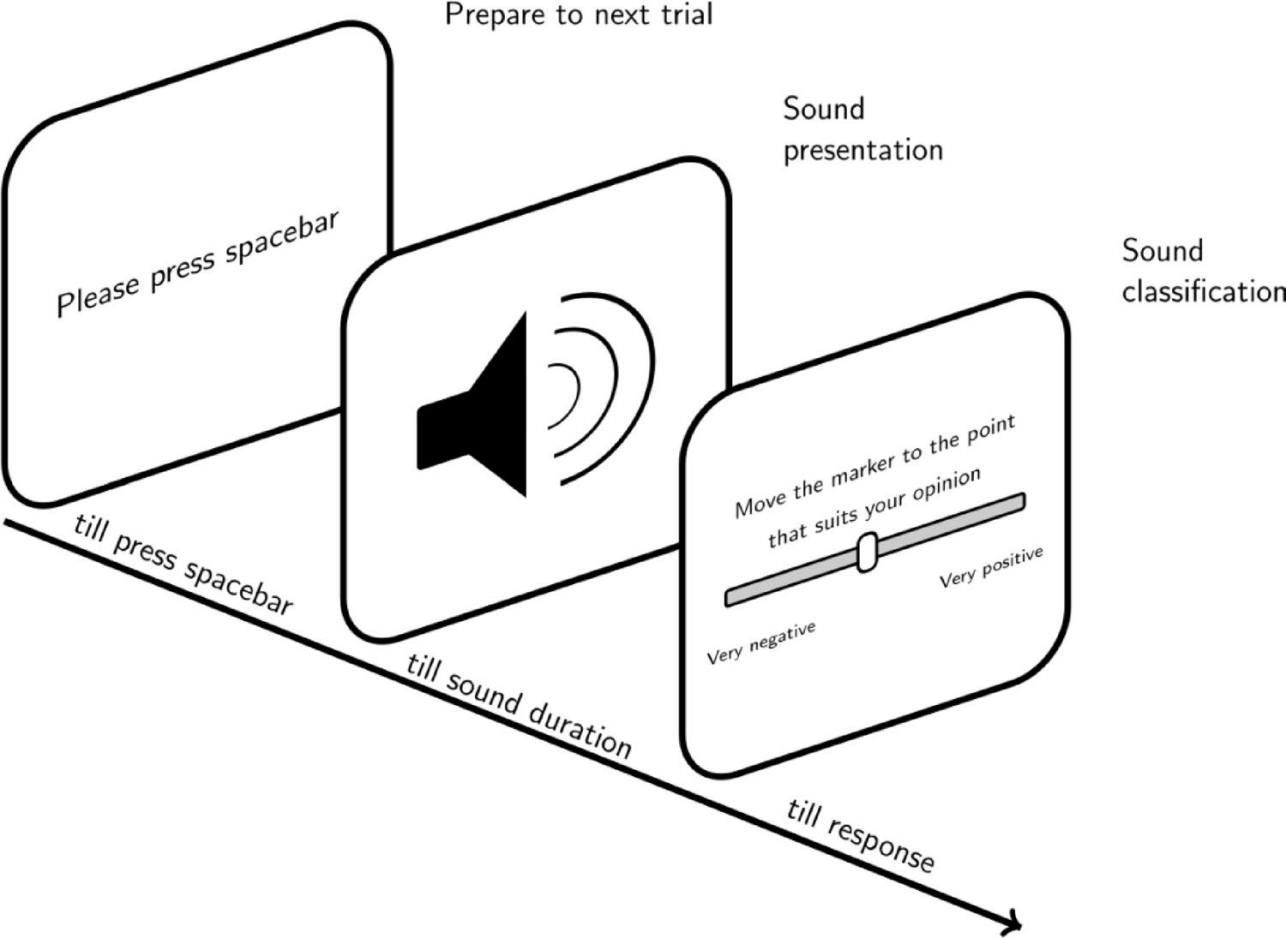
Sequence of events in Experiment 1

The control sound was a 1-second pink noise with a 5 ms fade in/out, chosen for its equal energy distribution across octave bands. All sounds were created in Audacity and calibrated using ArtemiS software to ensure uniform dBA levels. Each trial commenced with a visual prompt instructing participants to press the spacebar. Subsequently, the sound was presented, and then participants used a slider to indicate their valence rating (Fig. 1). At the end, the ratings for each sound were recorded.

#### Experiment 2—Explicit task

**Fig. 2:**
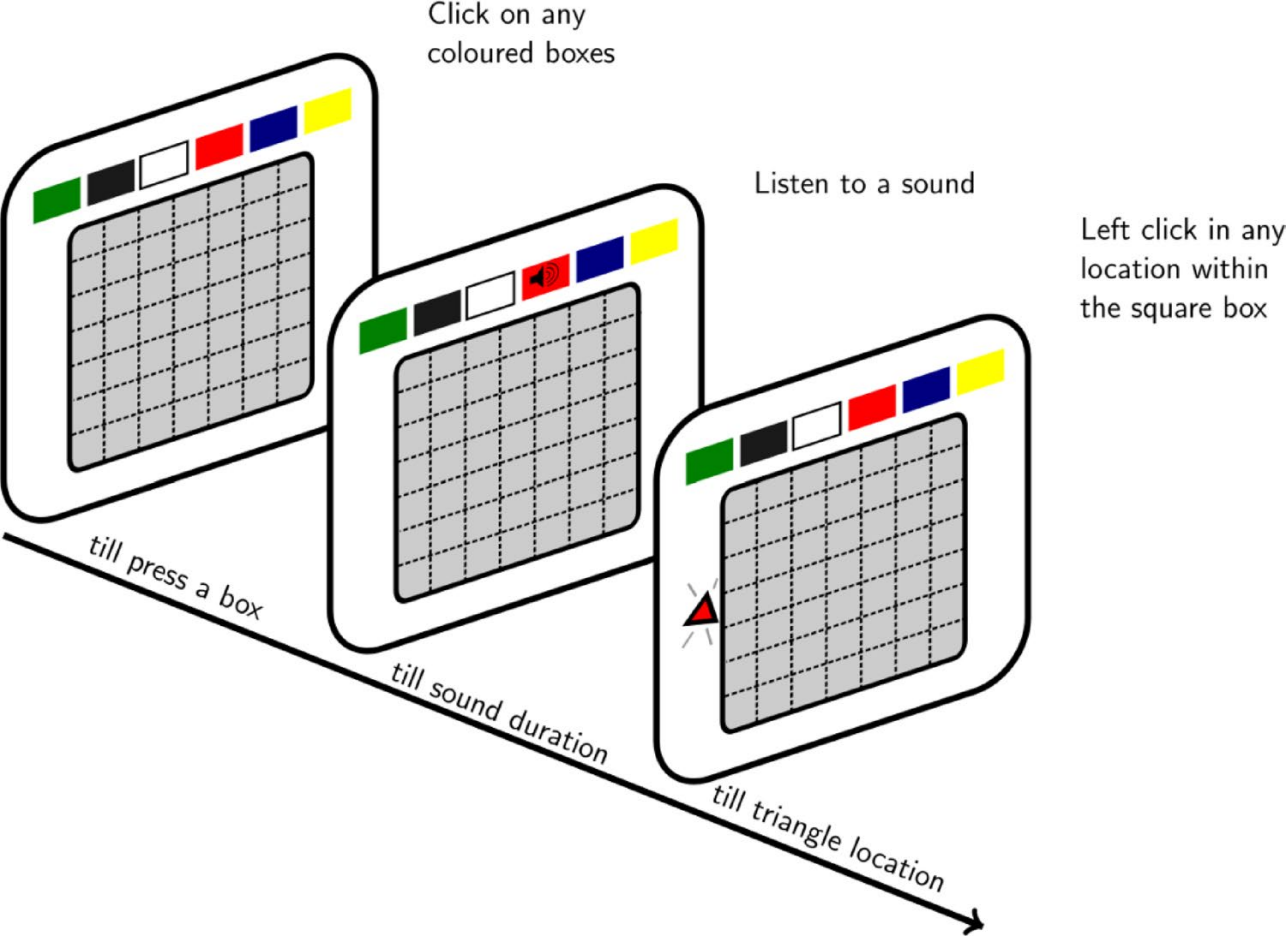
Sequence of events in Experiment 2. The illustration shows a mock trial in which the red box was clicked, the respective sound was produced, and then the red triangle was positioned within the square to be dragged onto it

Participants also listened to the same six auditory stimuli, one at a time, from Experiment 1 via headphones while sitting in front of a computer and positioning a triangle shape that represents the sound they heard within a two-dimensional square box. The experiment began with six colored boxes displayed at the top of the screen (Fig. 2). Participants were instructed to click on any box to hear a corresponding sound, allowing them to listen to the sounds in any order they liked. Subsequently, they were required to drag a geometric figure of the same color as the clicked colour box into a designated square box located beneath the colour boxes. Participants had the option to re-listen to the sound and adjust the figure’s position. Finally, the Cartesian coordinates of each geometric figure were recorded.

#### Experiment 3—Implicit task

**Fig. 3:**
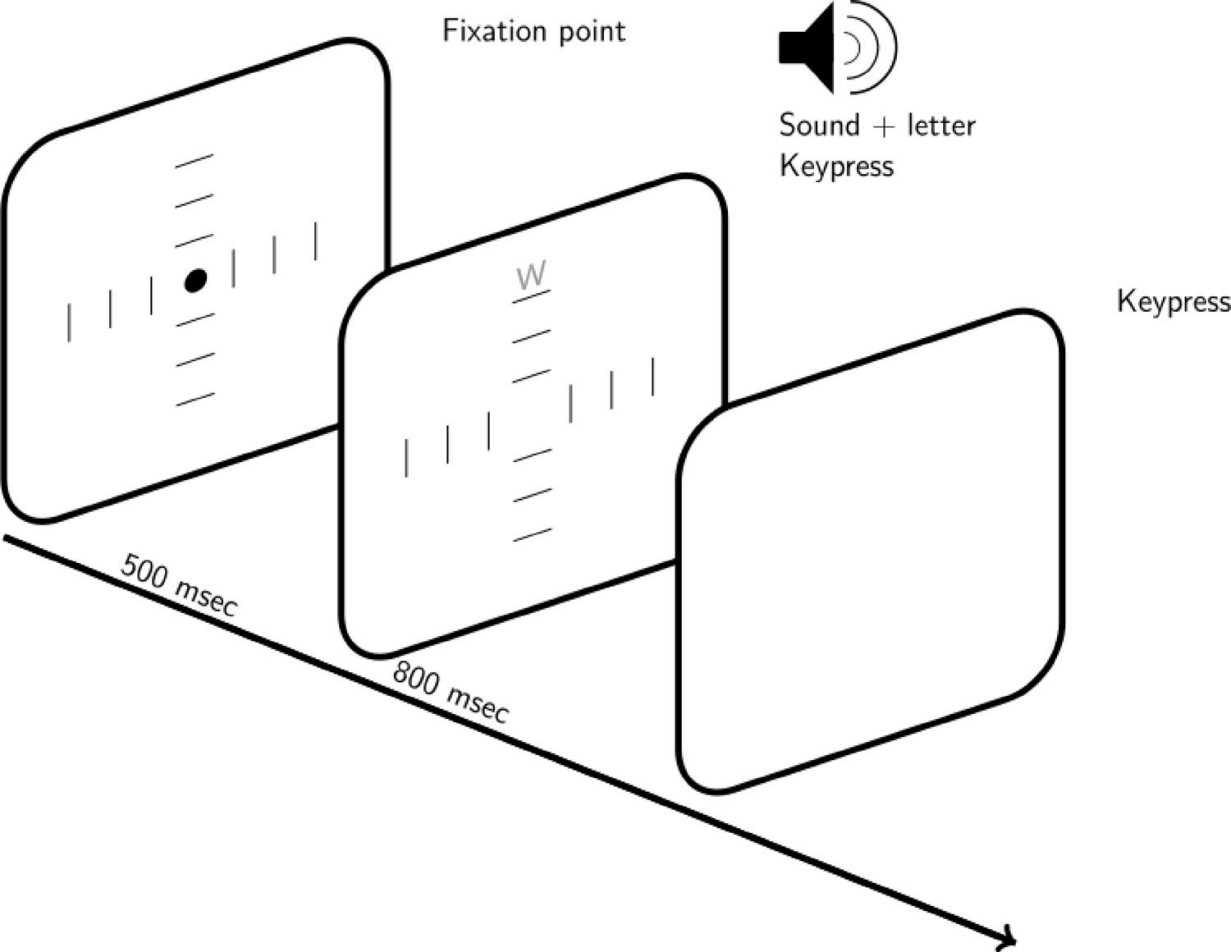
Sequence of events in Experiment 3. The illustration represents a mock trial wherein the letter W appears on the top uppermost location on the vertical axis of the screen for 800 ms. The trial terminates immediately if the participant responds before the stimulus offset; otherwise, a blank screen remains until a response is registered. The six vertical and six horizontal lines indicate where the letters could appear. These lines were not present during the task and are shown here for illustration purpose

This experiment required participants to quickly identify and respond to the letters ‘M’ or ‘W’ presented within a grid of horizontal and vertical lines (Fig. 3) on a computer screen. The target letters exhibited low contrast, closely resembling the screen gray background. Concurrently, one of six auditory stimuli from Experiment 1 was presented. Each trial began with a fixation point, followed by the simultaneous presentation of the sound and letter, and concluded with a speeded key press indicating the letter identified (Fig. 3). The responses recorded were reaction time and frequency of correct responses.

### Hypotheses

First, consistent with prior research (Rusconi et al. 2006; Lidji et al. 2007; Evans and Treisman 2010), a vertical crossmodal mapping of pitch (‘high pitch – high position / low pitch – low position’) should emerge both in the explicit spatial matching task (Experiment 2) and as a vertical congruency effect on reaction times in the implicit task (Experiment 3).

Second, a lateral crossmodal mapping of pitch (‘low pitch – left position / high pitch – right position’) should emerge in the explicit spatial matching task (Experiment 2), whereas the corresponding lateral congruency effect on reaction times in the implicit task (Experiment 3) should be weaker or absent, consistent with Rusconi et al. (2006) and Lidji et al. (2007).

Third, the lateral pitch-space mapping should be modulated by cross-national factors. Following Nishimura and Yokosawa (2009), the difference between the explicit task (Experiment 2) and the implicit task (Experiment 3) should be smaller in samples where metaphors describing pitch variation other than ‘high-low’ are more prevalent in the local language (Majid et al. 2018).

Fourth, based on Beecham et al. (2009) and Dolscheid and Casasanto (2015), we hypothesize that the crossmodal mappings between pitch and the vertical and horizontal spaces arise from a learning process involving the creation of independent mental representations. Consequently, the strength of the lateral pitch-space mapping at the individual participant level should be independent of the strength of the vertical pitch-space mapping.

Finally, Experiment 1 was exploratory in nature and tested whether pitch height maps onto emotional valence, and if so, whether this mapping is linear or departs from linearity at perceptual extremes (Gabrielsson and Lindström 2010; Scherer and Oshinsky 1977).

### Data analysis

Multilevel linear models were employed for data analysis using the *lmerTest*, *lme4* and *rstanarm* packages in R. Although each experiment was analyzed with a distinct model, all models included the following fixed factors: (a) laboratory location (categorical variable with three levels), (b) handedness (categorical variable with two levels), (c) gender (categorical variable with two levels), and (d) age (discrete numeric variable). Participant ID was included as a random factor. The Wilkinson-Rogers notation (Wilkinson and Rogers 1973) describing the model in each experiment are presented below.

**Experiment 1 (Model 1):** Rating was modeled as a function of presented sounds, laboratory location, gender, age, and participant ID (random effect). Here, ‘rating’ represents the valence scale value selected by the participant (continuous variable ranging from − 200 to 200; here called ‘v.rating’), ‘sounds’ represents the presented sounds (categorical variable with six levels), ‘lab’ represents the laboratory location, ‘gender’ and ‘age’ represent the participant’s gender and age, respectively, and ‘participant’ represents the participant ID.

1 $$ \begin{aligned} {\rm{v}}.{\rm{rating}} & \,\sim \,{\rm{sounds}}\, + \,{\rm{lab}}\, + \,{\rm{handedness }} \\& \quad + ~{\rm{gender}}\, + \,{\rm{age }} + {\rm{ }}\left( {{\rm{1}}|{\rm{participant}}} \right) \end{aligned} $$

**Experiment 2 (Models 2 and 3):** The Cartesian coordinates of the shape representing the sound in ‘x’ (horizontal) and ‘y’ (vertical) locations were modeled as a function of the same fixed factors as in Experiment 1, and with participant ID as a random effect.

2 $$ \begin{aligned} {\rm{x}} &\,\sim \,{\rm{sounds }} + ~{\rm{lab }} + ~{\rm{handedness }} \\& \quad + ~{\rm{gender}}\, + \,{\rm{age }} + {\rm{ }}\left( {{\rm{1 }}|{\rm{ participant}}} \right) \end{aligned} $$

3 $$ \begin{aligned} {\rm{y}} &\,\sim \,{\rm{sounds }} + ~{\rm{lab }} + ~{\rm{handedness }} \\& \quad + ~{\rm{gender}}\, + \,{\rm{age }} + {\rm{ }}\left( {{\rm{1 }}|{\rm{ participant}}} \right) \end{aligned} $$

**Experiment 3 (Models 4 and 5):** Reaction times for correct letter identification in vertical (‘RTv’) and horizontal (‘RTh’) positions were modeled as a function of letter position (categorical variable with six levels), laboratory location, gender, age, and participant ID. Here, ‘letter position’ represents the position of the letter on the screen. For this model, the main effects and the nteraction between letter position and sounds were evaluated.

4 $$ \begin{aligned} {\rm{RTv }} & \sim ~{\rm{letter \; position }}*{\rm{ sounds }} + ~{\rm{lab }} + ~{\rm{handedness }} \\& \quad + ~{\rm{gender}}\, + \,{\rm{age }} + {\rm{ }}\left( {{\rm{1 }}|{\rm{ participant}}} \right) \end{aligned} $$

5 $$ \begin{aligned} {\rm{RTh }} & \sim ~{\rm{letter \; position }}*{\rm{ sounds }} + ~{\rm{lab }} + ~{\rm{handedness }} \\& \quad + ~{\rm{gender}}\, + \,{\rm{age }} + {\rm{ }}\left( {{\rm{1 }}|{\rm{ participant}}} \right) \end{aligned} $$

We employed both frequentist and Bayesian modeling to provide a complementary assessment of our predictors, using *p*-values for long-term error control and Bayes Factors to quantify the relative evidence for our hypotheses (Schubert et al. 2025). For this comparison, we used the *lmer* function (from the *lme4* package) and the *stan_lmer* function (from the *rstanarm* package), respectively. In cases of congruent results, we chose the frequentist model for presentation (the Bayesian modelling is available in the R Supplementary Files). Furthermore, due to the unbalanced distribution of the handedness variable, we conducted a power analysis to determine whether it should be retained in the final model.

After identifying the best model according to these comparisons and power analysis, marginal and conditional R2 values were computed using the *r2_nakagawa* command from the *performance* R package. The variance components of the random factors were then estimated using the *gstudy* command from the *gtheory* R package.

Prior to our analysis, tests for normality and homoscedasticity were conducted using the *gvlma* R package to verify that the data distributions satisfied the assumptions of the linear models. A significance level of *p* < 0.05 was used for all statistical tests. Appropriate pairwise comparisons followed by a Holm–Bonferroni correction were performed when needed.

## Results

### Experiment 1

**Fig. 4:**
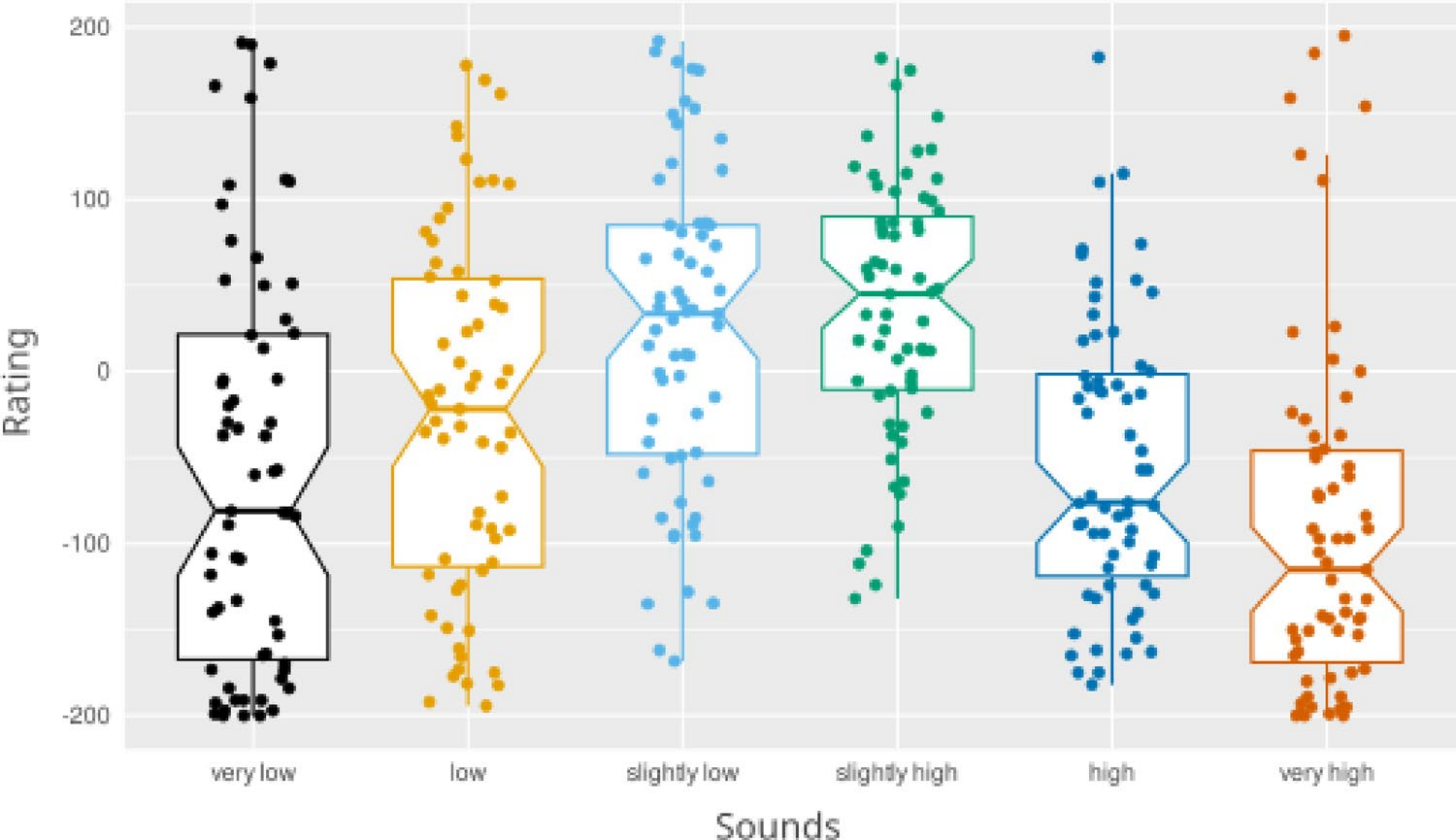
Results from Experiment 1 in which the median rating assigned to each of the six sounds is shown (notches represent ~ 95% confidence intervals around the median). Out of the 15 pairwise comparisons, five were non-significant (adjusted p-value; see Supplementary Materials for details). The more negative the median rating, the more negatively the sound was perceived. A curvilinearity analysis confirmed the inverted U pattern in this analysis (see Supplementary Materials). A model incorporating an interaction between ‘sound’ and ‘lab,’ while controlling for additional factors, revealed that specific sounds were rated differently across countries. Nevertheless, the overall average rating for each sound remained consistent across countries, as depicted in the figure (see Supplementary Materials)

The final model does not include the handedness variable, due to its low explainability power. Furthermore, as the data did not exhibit normal or homoscedastic distributions, a robust linear mixed model was employed (Koller 2016). After comparison with Bayesian models, the frequentist robust linear mixed models were selected to be presented as results. Table 2 presents the summary of this model for Experiment 1. Based on an intercept defined by the responses of participants from the Japanese lab who identified as left-handed women and were presented with a very low sound, it can be concluded that sounds in the middle of the spectrum were rated significantly more positive than the other sounds (Fig. 4). No other predictor demonstrated a significant difference compared to the intercept.

**Table 2:** Summary of the robust mixed linear model for valence ratings in Experiment 1

	Estimate	Std. error	t value	Significance level
(Intercept)	−40.605	29.199	−1.391	
Pitch low	40.076	16.998	2.359	
Pitch slightly low	92.812	16.998	5.43	**
Pitch slightly high	103.935	16.998	6.118	**
Pitch high	10.540	16.998	0.620	
Pitch very high	−37.237	16.998	−2.191	
Poland lab	19.730	15.909	1.240	
Spain lab	−1.445	17.313	−0.083	
Gender male	4.323	13.235	0.326	
Age	−1.536	1.240	−1.238	

Significant values based on the degree of freedom from the non-robust version of the same linear mixed model, were used as references to index statistical significance. Signif. codes: 0.001 ‘**’ 0.01 ‘*’

### Experiment 2

**Fig. 5:**
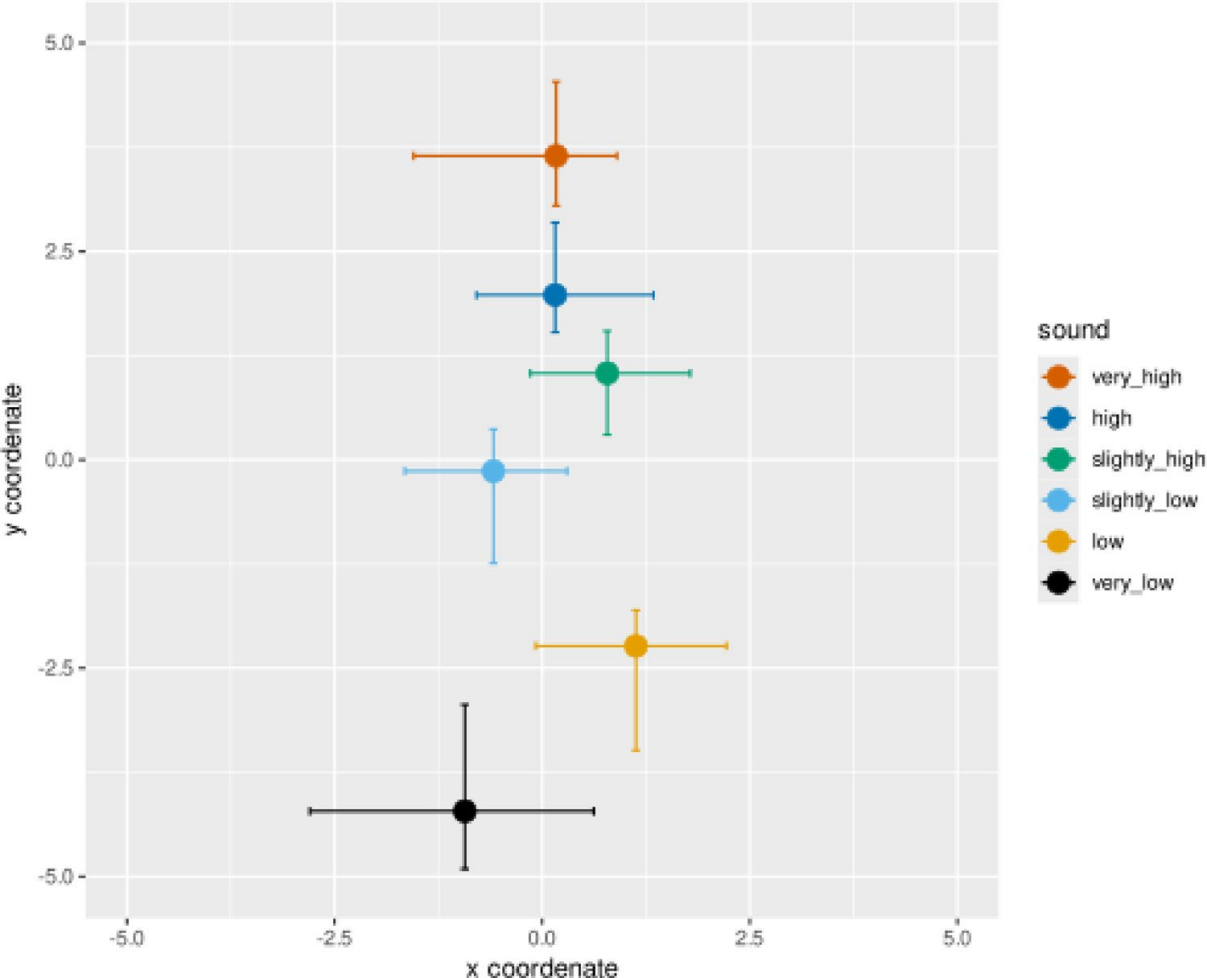
Plot displays the median localization (± 95% Bootstrap Confidence Interval for each sound) of the sounds within the square box in Experiment 2. The *x* and *y* coordinates represent the location in which each sound was located, within a range of values from − 5 to 5. A model incorporating an interaction between ‘sound’ and ‘lab,’ while controlling for additional factors, revealed that specific sounds were positioned differently along the Y-axis across countries (this did not occur on the X-axis). Nevertheless, the overall average rating for each sound remained consistent across countries, as depicted in the figure (see Supplementary Materials)

**Fig. 6:**
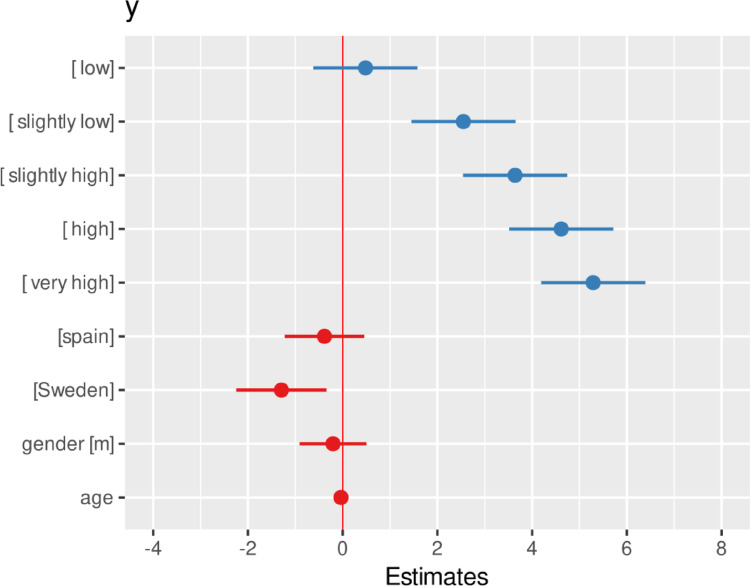
Forest plot for estimated values from the linear mixed model for the vertical data of the Experiment 2. The vertical red line represents the intercept (the responses participants from the Japanese lab who identified as left-handed women and were presented with a very low sound = −1.48), and the dots and horizontal lines represent the estimation of the difference between the predictor levels and the intercept (blue dot whiskers represent estimates above the intercept, while red dot whiskers represent estimates below the intercept). The average localization of a very low sound was associated with a negative *y*-coordinate. Specifically, a low sound (0.48) was localized slightly higher on the *y*-axis compared to a very low sound, resulting in a coordinate of −1.004 (−1.48 + 0.48). Conversely, a very high sound (5.28) was localized at a positive coordinate of 3.80 (−1.48 + 5.28). Error bars consistently depict 95% CIs across these localizations

Similar to Experiment 1, the handedness variable was excluded from the model due to its limited explanatory power. Furthermore, due to the horizontal coordinate data (x-coordinate) not exhibiting normal or homoscedastic distributions, a robust linear mixed model was employed. In contrast, a linear mixed model adequately fits the vertical coordinate data (y-coordinate). After comparisons between the robust and frequentist linear mixed models for the horizontal and vertical coordinates with their respective Bayesian version models, the linear models were selected to be presented as results. The robust model for the horizontal data revealed no significant differences between any factors (see Supplementary Materials). Conversely, the vertical model demonstrated significant differences for both sound and laboratory variables (Table 3). Specifically, based on an intercept defined by the responses of participants from the Japanese lab who identified as left-handed women and were presented with a very low sound, all other sounds were positioned at a significantly higher y-coordinate (Figs. 5, 6). Furthermore, participants from the Sweden lab positioned all sounds at a significantly lower y-coordinate compared to the intercept (Fig. 6).

**Table 3:** Analysis of deviance table (type III Wald χ^2^ tests) for the fixed effects of the model of vertical data of Experiment 2

	Chisq Df	Df	Pr(> Chisq)
(Intercept)	1.5121	1	0.2188
Sounds	149.8868	5	< 2e−16 ***
Laboratory	7.1656	2	0.0278*
Gender	0.3195	1	0.5719
Age	0.5481	1	0.4591

### Experiment 3

**Fig. 7:**
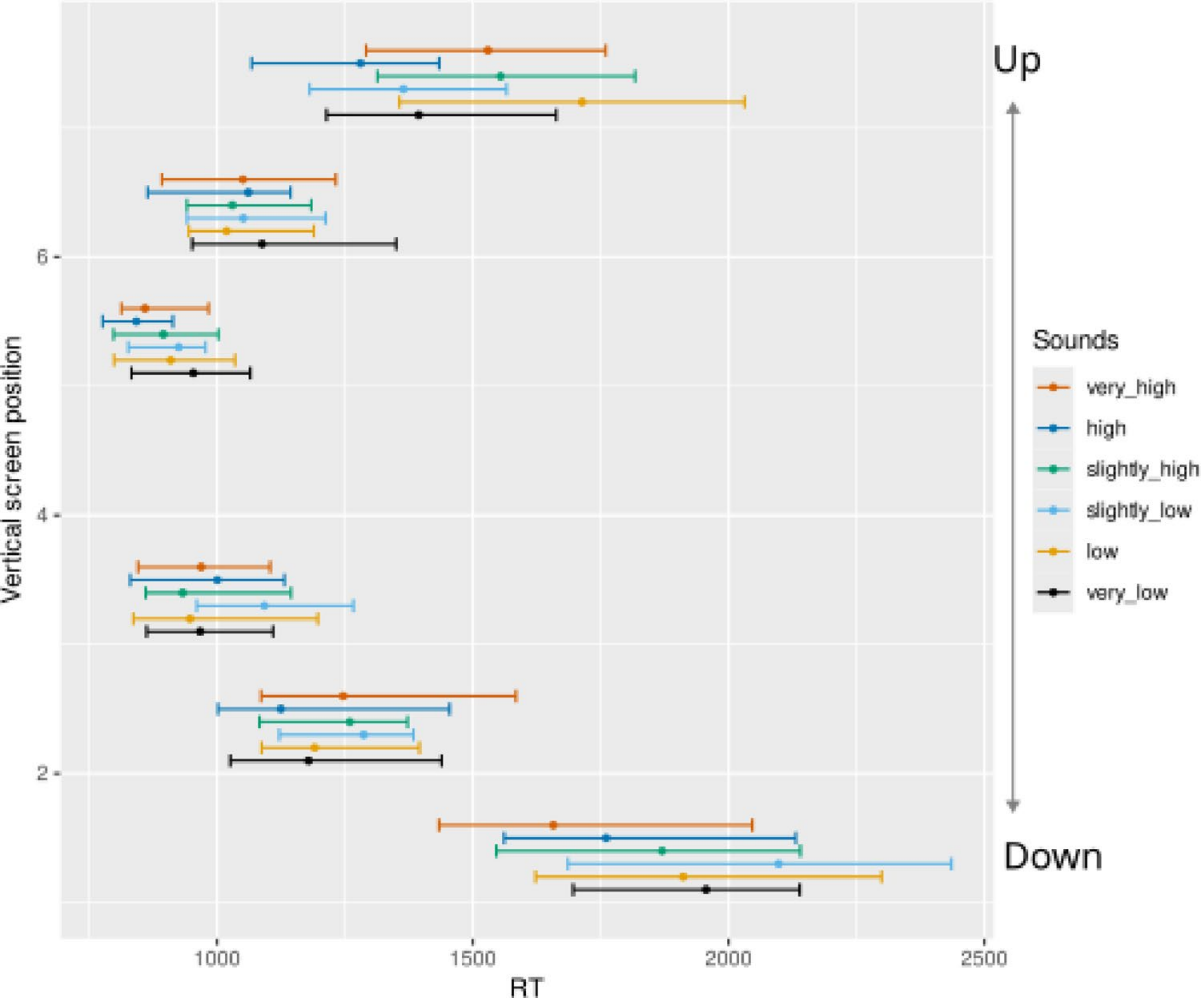
Dot plots representing the reaction times (x-axis) for the vertical task of Experiment 3 when the participants correctly identified the letter M or W presented at various vertical screen positions (y-axis). Error bars represent 95% CI

**Fig. 8:**
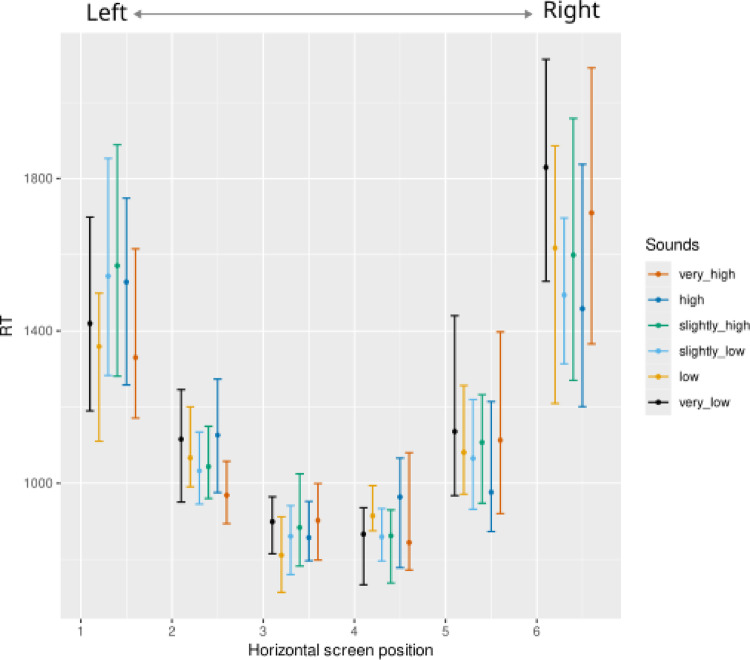
Dot plots representing the reaction (y-axis) times for the horizontal task of Experiment 3 when the participants correctly identified the letter M or W presented at various horizontal screen positions (x-axis). Error bars represent 95% CI

For experiment 3, as in the previous experiments, the handedness variable was not included in the model due to its low explainability power. Regarding the tests for normality and homoscedasticity, when participants correctly identified the letter presented in either the horizontal or vertical position, none of the RTs showed a normal or homoscedastic distribution. Thus, robust linear mixed models were again employed to analyze the Experiment 3 data. Nevertheless, none of the robust linear mixed models identified differences between the RTs, the position of the letter and the height of the presented pitch (see Supplementary Materials). Figures 7 and 8 show that the RTs increased with the distance from the center of the screen, the further away from the center, regardless of whether it appears in the vertical or horizontal position, the longer the RT. The type of sound did not have any relationship with the speed of identification of the target stimulus and their spatial location.

## Discussion

The primary aim of this study was to investigate the relationship between pitch height, spatial mapping, and affective evaluation across several countries. The results from Experiment 2 support established findings while also suggesting new insights, particularly regarding cross-national differences and the independence of vertical and horizontal mappings. By contrast, Experiment 1 revealed a new metaphor for describing pitch variation when an emotional dimension is included, displaying an inverted U pattern.

Across experiments, the vertical dimension emerged as the dominant axis for spatial representation of pitch height. In particular, the results of Experiment 2 clearly demonstrated that participants consistently localized higher-pitched sounds at higher spatial positions. The only exception is Swedish, where sounds are positioned slightly lower, confirming our third hypothesis, at least with regard to Experiment 2. This can be related to a specific characteristic of Swedish speakers, who sometimes use different metaphors for pitch to those used in other languages (Dolscheid et al. 2020; Majid et al. 2018). For example, Swedish describes high pitch as ‘ljus’ (light/bright) and low pitch as ‘mörk’ (dark). Furthermore, these results may have been influenced by the presence of participants with absolute pitch, who might not exhibit the behavioral patterns typically associated with pitch-variation metaphors (Spence and Di Stefano 2024).

The emergence of the vertical dimension confirms earlier research (Pratt 1930; Trimble 1934; Rusconi et al. 2006) showing a robust tendency to perceive higher pitches as “higher” in space—at least for Experiment 2. In this sense, the consistency of this effect observed across laboratories supports the hypothesis that this type of mapping may have a structural or statistical basis (Spence 2011; Dolscheid et al. 2014; Parkinson et al. 2012), as it has also been observed in infants and in speakers of linguistically diverse backgrounds. This notion also aligns with broader frameworks like A Theory of Magnitude (ATOM), which posits that a common neural metric underlies the perception of space and other quantities (Walsh 2003; Fabbri et al. 2012). This result partially confirms our first hypothesis. The vertical crossmodal mapping of pitch clearly emerged in the explicit spatial matching task (Experiment 2). However, no corresponding vertical congruency effect on reaction times was observed in the implicit task (Experiment 3); we return to this discrepancy below.

An additional noteworthy finding comes from Experiment 1, which revealed a non-linear relationship between pitch height and emotional valence: extremely high and extremely low tones were rated as significantly less positive compared to moderately high or low tones. Specifically, the extremely low tones (40–90 Hz) may elicit a sensation of ‘roughness’ or ‘beating.’ This occurs when the harmonics of simultaneous tones are insufficiently spaced, leading to waveform interference that the auditory system perceives as rough (Di Stefano and Spence 2022). Conversely, tones exceeding 2000 Hz are often characterized by ‘sharpness’ or a ‘hissing’ quality (Di Stefano and Spence 2022). Given that both roughness and sharpness are consistently correlated with increased annoyance and perceived harshness, this suggests that perceptual extremes may be emotionally less pleasant, reinforcing the idea that pitch height is a cognitively and affectively salient dimension.

In contrast to the clear confirmation of our first hypothesis regarding the vertical dimension, the horizontal mapping of pitch proved inconsistent and weak, thereby contradicting our second hypothesis. Experiment 2 revealed no systematic differences in the horizontal placement of tones, and Experiment 3 likewise showed no effect of pitch height on reaction times with respect to the horizontal position of the visual stimuli. These findings are consistent with previous studies (Rusconi et al. 2006; Lidji et al. 2007; Jaap and Rose 2024), which suggest that horizontal mapping is less automatic and likely more dependent on musical training or explicit attentional focus on pitch.

The null results from Experiment 3, however, warrant a deeper methodological consideration. This task was designed to capture implicit, automatic cross-modal mappings. While some studies (e.g., Evans and Treisman 2010; Parise and Spence 2012) suggest that these mappings are automatic and take place at the perceptual level, others indicate that they may be modulated by top-down factors, such as attention and cognitive load (Jaap and Rose 2024). The task required participants to identify low-contrast letters (‘M’ or ‘W’), which places a higher demand on focal attention than the process described by Evans and Treisman (2010), who worked with geometric shapes rather than letters, since the symbolic nature of letter identification may simply be too distant from the perceptual mapping of pitch to trigger an interference effect under stress.

The high cognitive load of this task could have overshadowed or suppressed the processing of the auditory stimuli, which were entirely irrelevant to the task’s objective. Second, the complete lack of predictive value of the sounds for the letter’s position or identity might have led participants to actively filter them out as noise. For an implicit association to manifest, the irrelevant dimension (pitch) may need to be perceived as at least peripherally related to the task at hand, which was not the case here. Consequently, the absence of an effect in this specific paradigm does not definitively rule out the existence of an automatic horizontal mapping, but rather highlights the conditions under which it may not be observable.

A partial exception to this pattern can be found in the study by Nishimura and Yokosawa (2009), who observed a congruency effect between pitch height and horizontal space even among Japanese participants with no formal musical training. The authors attributed this finding to the comparatively intensive musical education provided in Japanese schools. This cultural difference was directly tested in our study through cross-national data collection. Although horizontal pitch-space mapping did not emerge in either Experiment 2 or the more automatic task of Experiment 3, it should be noted that capturing subtle, country-contingent effects may depend strongly on task type and methodological design. Further research is therefore warranted to explore these cultural modulations in more depth. Understanding such group differences has become an area of growing interest within the study of cross-modal correspondences (Spence 2022).

One of the central theoretical aims of this study was to compare two competing accounts of spatial-pitch mapping: the Mental Metaphor Theory (Casasanto 2013) and the Polarity Correspondence Hypothesis (Proctor and Cho 2006). Our findings favor the Mental Metaphor Theory, which posits that different conceptual metaphors are represented independently. Specifically, no relationship was found between the magnitude of the crossmodal mappings on the vertical and horizontal axes, which did not confirm our fourth hypothesis. Experiment 2 offers evidence consistent with prior research by Beecham et al. (2009) and Dolscheid and Casasanto (2015). These studies similarly demonstrated the independent operation of various spatial-pitch correspondences, such as pitch-height versus pitch-thickness or number-space mappings. Such independence lends support to recent views suggesting that different correspondences can have distinct origins—be they statistical, semantic, or affective—and thus do not necessarily rely on a single, unified mechanism (Motoki et al. 2023). These results do not support the integrative mechanism proposed by the Polarity Correspondence Hypothesis, which would predict a shared source of congruency effects across dimensions.

### Constraints on generality

As Simons et al. (2017) suggest, no study is free from limitations, and this study is no exception. First, although our sample included a relatively high number of left-handed participants, the role of handedness and lateralization in spatial-pitch associations remains unclear. This is especially relevant in light of the “octave illusion” (Deutsch 1974; Oehler and Reuter 2013), which may interact with individual lateralization patterns. Although handedness was included as a factor in all statistical models, it did not emerge as a significant predictor. Future studies with balanced samples of left- and right-handed individuals could help clarify whether manual dominance systematically modulates crossmodal mappings.

A second and significant limitation is the absence of data on participants’ musical background and training. The introduction repeatedly cites musical expertise as a key potential modulator of horizontal pitch-space mappings, a factor that could explain discrepancies in previous literature (e.g., Nishimura and Yokosawa 2009). By not assessing this variable, we are unable to disentangle the potential effects of formal musical training from broader cultural influences. This omission weakens the interpretation of our cross-national comparisons, as any observed differences (or lack thereof) could be confounded by varying levels of musical expertise within and between the sampled populations. Future research aiming to investigate cultural modulations of pitch mapping must incorporate a standardized measure of musical experience to control for this crucial variable.

A third limitation concerns the selection of auditory stimuli, which were ‘flat’ relative to standard Western tuning. This characteristic may also influence the generalizability of the findings, particularly regarding musically trained individuals who are accustomed to standard pitch categories.

Finally, for future research, it would be worthwhile to explore advanced regression techniques, such as those utilizing copulas to model the dependence between the outcomes. This could reveal interesting insights on how the association varies according to specific covariates. A preliminary analysis with data from Experiment 1 employed distributional copula regression (Klein and Kneib 2016), a method we plan to investigate further in our future studies with regard to interpretation and model building.

## Data Availability

Stimuli, data, and R codes are available at https://cutt.ly/wrxtOnk4.

## Footnotes

1 It is worth noting that the relationship between pitch and affect is also modulated by musical mode, which concerns pitch relationships within scales and keys. A substantial body of research has demonstrated that the major mode is generally associated with happiness and positive affect, whereas the minor mode tends to evoke sadness and negative affect (Hevner 1935; Crowder 1984; Kastner and Crowder 1990; Dalla Bella et al. 2001). These mode–emotion associations appear early in development and have been observed cross-culturally (Fritz et al. 2009). Since the present study employed isolated complex tones rather than melodic or harmonic sequences, mode-related affective influences were not directly relevant to our stimuli. Nevertheless, the broader interplay between pitch height and mode in shaping emotional responses underscores the multidimensional nature of pitch–affect correspondences.

2 It should be noted that the term “pitch height” as used throughout this manuscript refers to the continuous, monotonically increasing dimension of pitch perception that corresponds to fundamental frequency, as distinguished from “pitch chroma”—the cyclical quality of pitch that repeats at octave intervals (Bachem 1950). While our stimulus spacing resulted in variations of both pitch height and pitch chroma, the cross-modal correspondences under investigation here (pitch–elevation, pitch–laterality) are theoretically grounded in the pitch height dimension rather than chroma. A spacing in octave intervals would have equalized chroma across stimuli but would have severely constrained the number of perceptually distinguishable pitch levels within the audible range. Finally, it is important to acknowledge that the stimuli were ‘flat’ relative to standard Western tuning.

## Code availability

Stimuli, data, and R codes are available at https://cutt.ly/wrxtOnk4.

## References

[CR1] Bachem A (1950) Tone height and tone chroma as two different pitch qualities. Acta Psychol 7:80–88. 10.1016/0001-6918(50)90004-7

[CR2] Beecham R, Reeve RA, Wilson SJ (2009) Spatial representations are specific to different domains of knowledge. PLoS ONE 4(5):e5543 19461994 10.1371/journal.pone.0005543 PMC2678257

[CR3] Bernstein IH, Edelstein BA (1971) Effects of some variations in auditory input upon visual choice reaction time. J Exp Psychol 87(2):241–247 5542226 10.1037/h0030524

[CR4] Bregman AS, Steiger H (1980) Auditory streaming and vertical localization: Interdependence of what and where decisions in audition. Percept Psychophys 28(6):539–546 7208267 10.3758/bf03198822

[CR5] Casasanto D (2009) Embodiment of abstract concepts: good and bad in right- and left-handers. J Exp Psychol Gen 138(3):351–367. 10.1037/a0015854 19653795 10.1037/a0015854

[CR6] Casasanto D (2013) Development of metaphorical thinking: the role of language. In: Borkent M, Dancygier B, Hinnell J (eds) Language and the creative mind. CSLI, Stanford, CA, pp 3–18

[CR7] Chiou R, Rich A (2012) Cross-modality correspondence between pitch and spatial location modulates attentional orienting. Perception 41:339–353. 10.1068/p7161 22808586 10.1068/p7161

[CR8] Cho YS, Bae GY, Proctor RW (2012) Referential coding contributes to the horizontal SMARC effect. J Exp Psychol Hum Percept Perform 38(3):726–734 22141586 10.1037/a0026157

[CR9] Crowder RG (1984) Perception of the major/minor distinction: I. Historical and theoretical foundations. Psychomusicology 4(1–2):3–12

[CR10] Dalla Bella S, Peretz I, Rousseau L, Gosselin N (2001) A developmental study of the affective value of tempo and mode in music. Cognition 80(3):B1–B10. 10.1016/S0010-0277(00)00136-0 11274986 10.1016/s0010-0277(00)00136-0

[CR11] Dehaene S, Bossini S, Giraux P (1993) The mental representation of parity and number magnitude. J Exp Psychol Gen 122:371–396

[CR12] Deutsch D (1974) An auditory illusion. Nature 251:307–309 4427654 10.1038/251307a0

[CR13] Di Stefano N (2022) The spatiality of sounds: from sound-source localization to musical spaces. Aisthesis 15(1):173–185. 10.36253/Aisthe-sis-13617

[CR14] Di Stefano N, Spence C (2022) Roughness perception: a multisensory/crossmodal perspective. Atten Percept Psychophys 84(7):2087–2114. 10.3758/s13414-022-02550-y 36028614 10.3758/s13414-022-02550-y PMC9481510

[CR15] Dolscheid S, Casasanto D (2015) Spatial congruity effects reveal metaphorical thinking, not polarity correspondence. Front Psychol 6:1836 26635713 10.3389/fpsyg.2015.01836 PMC4659873

[CR19] Dolscheid S, Shayan S, Casasanto D, Majid A (2013) The thickness of musical pitch: psychophysical evidence for linguistic relativity. Psychol Sci 24(5):613–621 23538914 10.1177/0956797612457374

[CR18] Dolscheid S, Hunnius S, Casasanto D, Majid A (2014) Prelinguistic infants are sensitive to space-pitch associations found across cultures. Psychol Sci 25(6):1256–1261 24899170 10.1177/0956797614528521

[CR16] Dolscheid S, Çelik S, Erkan H, Küntay A, Majid A (2020) Space-pitch associations differ in their susceptibility to language. Cognition 196:104073. 10.1016/j.cognition.2019.104073 31810048 10.1016/j.cognition.2019.104073

[CR17] Dolscheid S, Çelik S, Erkan H, Küntay A, Majid A (2023) Children’s associations between space and pitch are differentially shaped by language. Dev Sci 26(5):e13341. 10.1111/desc.13341 36315982 10.1111/desc.13341

[CR20] Evans KK, Treisman A (2010) Natural cross-modal mappings between visual and auditory features. J Vis 10(1):6:1–12 20143899 10.1167/10.1.6 PMC2920420

[CR21] Fabbri M, Cancellieri J, Natale V (2012) The A Theory Of Magnitude (ATOM) model in temporal perception and reproduction tasks. Acta Psychol 139(1):111–123. 10.1016/j.actpsy.2011.09.006 10.1016/j.actpsy.2011.09.006 22000733

[CR22] Fernandez-Prieto I, Spence C, Pons F, Navarra J (2017) Does language influence the vertical representation of auditory pitch and loudness? i-Perception 8(3):2041669517716183 28694959 10.1177/2041669517716183 PMC5484432

[CR23] Fritz T, Jentschke S, Gosselin N, Sammler D, Peretz I, Turner R, Friederici AD, Koelsch S (2009) Universal recognition of three basic emotions in music. Curr Biol 19(7):573–576. 10.1016/j.cub.2009.02.058 19303300 10.1016/j.cub.2009.02.058

[CR24] Gabrielsson A, Lindström E (2010) The role of structure in the musical expression of emotions. In: Juslin PN, Sloboda JA (eds) Handbook of music and emotion: theory, research, applications. Oxford University Press, pp 367–400

[CR25] Geronazzo M, Avanzini F, Grassi M (2015) Absence of modulatory action on haptic height perception with musical pitch. Front Psychol 6:1369 26441745 10.3389/fpsyg.2015.01369 PMC4566038

[CR26] Gundlach RH (1935) Factors determining the characterization of musical phrases. Am J Psychol 47:624–644

[CR28] Hevner K (1935) The affective character of the major and minor modes in music. Am J Psychol 47(1):103–118

[CR27] Hevner K (1937) The affective value of pitch and tempo in music. Am J Psychol 49:621–630

[CR29] Ilie G, Thompson WF (2006) A comparison of acoustic cues in music and speech for three dimensions of affect. Music Percept 23(4):319–329

[CR30] Jaap C, Rose M (2024) Dissociable neuronal mechanism for different crossmodal correspondence effects in humans. Acta Neurobiol Exp 84(2):136–152. 10.55782/ane-2024-2439 10.55782/ane-2024-2439 39087840

[CR31] Juslin PN, Laukka P (2003) Communication of emotions in vocal expression and music performance: different channels, same code? Psychol Bull 129(5):770–814 12956543 10.1037/0033-2909.129.5.770

[CR32] Juslin PN, Sloboda JA (2010) Handbook of music and emotion: theory, research, applications. Oxford University Press

[CR33] Kastner MP, Crowder RG (1990) Perception of the major/minor distinction: IV. Emotional connotations in young children. Music Percept 8(2):189–201

[CR34] Klein N, Kneib T (2016) Simultaneous inference in structured additive conditional copula regression models: a unifying Bayesian approach. Stat Comput 26(4):841–860. 10.1007/s11222-015-9573-6

[CR35] Koller M (2016) robustlmm: an R package for robust estimation of linear mixed-effects models. J Stat Softw 75(6):1–24. 10.18637/jss.v075.i06 32655332 10.18637/jss.v075.i01 PMC7351245

[CR36] Küssner MB, Tidhar D, Prior HM, Leech-Wilkinson D (2014) Musicians are more consistent: gestural cross-modal mappings of pitch, loudness and tempo in real-time. Front Psychol 5:789 25120506 10.3389/fpsyg.2014.00789 PMC4112934

[CR37] Lega C, Cattaneo Z, Merabet LB, Vecchi T, Cucchi S (2014) The effect of musical expertise on the representation of space. Front Hum Neurosci 8:250 24795605 10.3389/fnhum.2014.00250 PMC4006044

[CR38] Lemaitre G, Scurto H, Françoise J, Bevilacqua F, Houix O, Susini P (2017) Rising tones and rustling noises: metaphors in gestural depictions of sounds. PLoS ONE 12(7):e0181786 28750071 10.1371/journal.pone.0181786 PMC5547699

[CR39] Lidji P, Kolinsky R, Lochy A, Morais J (2007) Spatial associations for musical stimuli: a piano in the head? J Exp Psychol Hum Percept Perform 33:1189–1207 17924817 10.1037/0096-1523.33.5.1189

[CR40] Majid A, Roberts SG, Cilissen L, Emmorey K, Nicodemus B, O’Grady L, Woll B, LeLan B, de Sousa H, Cansler BL, Shayan S, de Vos C, Senft G, Enfield NJ, Razak RA, Fedden S, Tufvesson S, Dingemanse M, Ozturk O, Levinson SC (2018) Differential coding of perception in the world’s languages. Proc Natl Acad Sci 115(45):11369–11376. 10.1073/pnas.1720419115 30397135 10.1073/pnas.1720419115 PMC6233065

[CR41] McCormick K, Lacey S, Stilla R, Nygaard L, Sathian K (2018) Neural basis of the crossmodal correspondence between auditory pitch and visuospatial elevation. Neuropsychologia 112:19–30. 10.1016/j.neuropsychologia.2018.02.029 29501792 10.1016/j.neuropsychologia.2018.02.029 PMC7838671

[CR42] Melara RD, Marks LE (1990) Processes underlying dimensional interactions: correspondences between linguistic and nonlinguistic dimensions. Mem Cogn 18:477–495 10.3758/bf03198481 2233261

[CR43] Motoki K, Marks LE, Velasco C (2023) Reflections on cross-modal correspondences: current understanding and issues for future research. Manuscript submitted for publication 10.1163/22134808-bja10114 37963487

[CR44] Mudd SA (1963) Spatial stereotypes of four dimensions of pure tone. J Exp Psychol 66:347–352 14051851 10.1037/h0040045

[CR45] Nishimura A, Yokosawa K (2009) Effects of laterality and pitch height of an auditory accessory stimulus on horizontal response selection: the Simon effect and the SMARC effect. Psychon Bull Rev 16:666–670 19648450 10.3758/PBR.16.4.666

[CR46] Oehler M, Reuter C (2013) The octave illusion and handedness: a replication of Deutsch’s 1974 study. Musicae Sci 17(3):277–289

[CR48] Parise CV, Spence C (2012) Audiovisual crossmodal correspondences and sound symbolism: a study using the implicit association test. Exp Brain Res 220(3–4):319–333. 10.1007/s00221-012-3140-6 22706551 10.1007/s00221-012-3140-6

[CR47] Parise C, Knorre V, Ernst K, O. M (2014) Natural auditory scene statistics shapes human spatial hearing. Proc Natl Acad Sci 111(16):6104–6108. 10.1073/pnas.1322705111 24711409 10.1073/pnas.1322705111 PMC4000839

[CR49] Parkinson C, Kohler PJ, Sievers B, Wheatley T (2012) Associations between auditory pitch and visual elevation do not depend on language: evidence from a remote population. Perception 41:854–861 23155736 10.1068/p7225

[CR50] Parrott S, Guzman-Martinez E, Ortega L, Grabowecky M, Huntington MD, Suzuki S (2015) Direction of auditory pitch-change influences visual search for slope from graphs. Perception 44(7):764–778 26541054 10.1177/0301006615596904 PMC4638167

[CR51] Pratt CC (1930) The spatial character of high and low tones. J Exp Psychol 13(3):278–285

[CR52] Proctor RW, Cho YS (2006) Polarity correspondence: a general principle for performance of speeded binary classification tasks. Psychol Bull 132:416–442 16719568 10.1037/0033-2909.132.3.416

[CR53] Repp BH, Knoblich G (2007) Action can affect auditory perception. Psychol Sci 18(1):6–7 17362369 10.1111/j.1467-9280.2007.01839.x

[CR54] Roffler SK, Butler RA (1968) Localization of tonal stimuli in the vertical plane. J Acoust Soc Am 43:1260–1266 5659494 10.1121/1.1910977

[CR55] Rusconi E, Kwan B, Giordano BL, Umiltà C, Butterworth B (2006) Spatial representation of pitch height: the SMARC effect. Cognition 99:113–129 15925355 10.1016/j.cognition.2005.01.004

[CR56] Santiago J, Román A, Ouellet M (2011) Flexible foundations of abstract thought: a review and a theory. In: Maas A, Schubert T (eds) Spatial dimensions of social thought. Mouton de Gruyter, Berlin, pp 41–110

[CR57] Scherer KR, Oshinsky JS (1977) Cue utilization in emotion attribution from auditory stimuli. Motivation Emot 1(4):331–346

[CR58] Schubert A-L, Steinhilber M, Kang H, Quintana DS (2025) Improving statistical reporting in psychology. Commun Psychol 3(1):156. 10.1038/s44271-025-00356-w 41238797 10.1038/s44271-025-00356-w PMC12618885

[CR59] Simons DJ, Shoda Y, Lindsay DS (2017) Constraints on Generality (COG): a proposed addition to all empirical papers. Perspect Psychol Sci 12(6):1123–1128. 10.1177/1745691617708630 28853993 10.1177/1745691617708630

[CR60] Spence C (2011) Crossmodal correspondences: a tutorial review. Atten Percept Psychophys 73:971–995 21264748 10.3758/s13414-010-0073-7

[CR61] Spence C (2022) Exploring group differences in the crossmodal correspondences. Multisens Res 35(5):495–536. 10.1163/22134808-bja10079 35985650 10.1163/22134808-bja10079

[CR62] Trimble OC (1934) Localization of sound in the anterior posterior and vertical dimensions of auditory space. Br J Psychol 24:320–334

[CR63] Walsh V (2003) A theory of magnitude: common cortical metrics of time, space and quantity. Trends Cogn Sci 7(11):483–488. 10.1016/j.tics.2003.09.002 14585444 10.1016/j.tics.2003.09.002

[CR64] Wilkinson GN, Rogers CE (1973) Symbolic description of factorial models for analysis of variance. J Roy Stat Soc Ser C 22:392–399

[CR65] World Medical Association (2013) Declaration of Helsinki: ethical principles for medical research involving human subjects. JAMA 310(20):2191–2194 24141714 10.1001/jama.2013.281053

[CR66] Di Stefano N, Spence C (2024) Should absolute pitch be considered as a unique kind of absolute sensory judgment in humans? A systematic and theoretical review of the literature. Cognition 249:105805. 10.1016/j.cognition.2024.105805 10.1016/j.cognition.2024.105805 38761646

